# Balancing work and motherhood: nurse-mothers’ exclusive breastfeeding experiences in rural northern Ghana

**DOI:** 10.3389/fgwh.2026.1706240

**Published:** 2026-03-12

**Authors:** Barman Laribick Dujin, Joseph Lasong, Emmanuel Asiedu Agyeman, Fausta Adjoawubong, Vincentia Attah, Amidu Alhassan, Yula Salifu

**Affiliations:** 1 Department of Population and Reproductive Health, School of Public Health, University for Development Studies, Tamale, Ghana; 2 Ghana Health Service, Tamale Regional Hospital, Tamale, Ghana; 3 Ghana Health Service, Savelugu Municipal Hospital, Savelugu, Ghana; 4 Ghana Health Service, Tamale West Hospital, Tamale, Ghana; 5 Department of Adult Health, School of Nursing and Midwifery, University of Cape Coast, Cape Coast, Ghana; 6 Department of Population, Family and Reproductive Health, School of Public Health, University of Ghana, Legon, Ghana

**Keywords:** exclusive breastfeeding, experiences, Ghana, nurses, perceptions, rural

## Abstract

**Introduction:**

Exclusive breastfeeding (EBF) for the first six months is an evidence-based intervention to reduce child morbidity and mortality. Nurses play a critical role as promoters and role models for EBF; yet little is known about their personal perceptions and experiences, especially in rural Northern Ghana. This study explored nurse-mothers’ perceptions and lived experiences of EBF in Savelugu Municipality, Northern Ghana.

**Methods:**

A qualitative exploratory design was used. Fifteen nurses who had delivered within the prior two years were purposively sampled. Semi-structured interviews were conducted between April–June 2023, audio recorded, and transcribed verbatim. Braun & Clarke's thematic analysis guided coding and theme development. Trustworthiness was supported through prolonged engagement, member checking, and an audit trail.

**Results:**

Three overarching themes emerged: (1) Perceptions of EBF: nurses had strong knowledge and positive attitudes toward EBF, though concerns about time, privacy, and work conflicts were evident; (2) Experiences of Practicing EBF: while most participants practiced EBF immediately postpartum, many discontinued or shifted to mixed feeding before six months upon returning to work; (3) Coping Strategies and Systemic Recommendations: participants relied on expression, night feeding, and family support when available, and suggested workplace lactation rooms, colleague support, family education, and longer maternity leave. These findings highlight the tension between professional knowledge/attitudes and structural realities such as short maternity leave and lack of lactation facilities.

**Conclusions:**

Nurse-mothers value EBF but face substantial structural and social barriers that limit sustained adherence. Strengthening maternity leave policies, workplace lactation support, and family-inclusive education are essential to help nurses and working mothers more broadly meet EBF recommendations.

## Introduction

Exclusive breastfeeding (EBF), defined as feeding infants only breast milk with no additional foods or liquids except prescribed medicines for the first six months of life, remains one of the most effective public health interventions for improving child survival (1–3). Globally, optimal breastfeeding practices could prevent an estimated 823,000 child deaths annually (2). In sub-Saharan Africa, where infant morbidity and mortality remain disproportionately high, the World Health Organization (WHO) identifies EBF as a cornerstone of child survival strategies. Despite sustained global advocacy, adherence to EBF remains suboptimal. Approximately 43% of infants under six months are exclusively breastfed worldwide (4). In Ghana, the 2022 Ghana Demographic and Health Survey reported a decline in EBF prevalence from 52% in 2014 to 43% in 2022, with pronounced regional disparities (5). Evidence consistently links maternal employment, socio-cultural norms, time constraints, and limited institutional support to early cessation of EBF (6–8).

A growing body of research has examined breastfeeding among working mothers, including nurse-mothers and midwives, across diverse settings. Studies from both high-income and low- and middle-income countries (LMICs) document common barriers such as short maternity leave, shift work, high workloads, inadequate workplace lactation facilities, and unsupportive organisational cultures (9–16). Parallel literature also demonstrates that nurses’ and midwives’ personal beliefs, knowledge, and experiences influence how they counsel and support breastfeeding women in clinical practice (6, 9–13). However, these strands of research are often treated separately: nurse-mothers are examined either as breastfeeding women navigating employment, or as health professionals promoting EBF, with limited integration of these dual identities.

Conceptually, nurses’ breastfeeding experiences can be understood through the lens of work–family conflict theory, which highlights how incompatible demands between paid work and family roles generate strain and constrain health-related behaviours (17, 18). From an organisational support for breastfeeding perspective, institutional policies, workplace infrastructure, and managerial norms critically shape whether knowledge and motivation translate into sustained practice (19, 20). Additionally, a gendered labour perspective underscores how breastfeeding responsibilities intersect with the feminisation of nursing, moral expectations of caregiving, and unequal distributions of reproductive labour (21, 22). Together, these frameworks illuminate a central paradox: nurses possess high professional knowledge and are expected to champion exclusive breastfeeding (EBF), yet they operate within organisational and social structures that systematically undermine their own ability to practise it (12, 13, 22). In Ghana, empirical evidence on nurses’ own breastfeeding experiences remains limited, particularly in rural and northern settings where health system constraints, socio-cultural norms, and gendered expectations may be more pronounced (23, 24). Existing studies have largely focused on EBF prevalence among the general population or on nurses’ knowledge and counselling practices, with little attention to how nurses’ personal breastfeeding experiences interact with their professional identities as health educators and role models. This represents both a contextual gap (rural Northern Ghana) and a conceptual gap (the under-theorised dual-role tension between professional expertise and constrained maternal practice).

This study addresses these gaps by qualitatively exploring the perceptions and lived experiences of nurse-mothers regarding exclusive breastfeeding in the Savelugu Municipality of Northern Ghana. While the primary analytic focus is on nurses’ personal breastfeeding experiences, these experiences are explicitly interpreted within the context of their professional role as health workers who promote EBF. By foregrounding the paradox between knowledge and practice, the study contributes novel, context-specific insights into how structural, organisational, and gendered constraints shape breastfeeding among health professionals in LMIC settings. The findings have implications for workplace breastfeeding support policies, health system governance, and broader efforts to align maternal employment conditions with national EBF goals and the Sustainable Development Goals related to child health (SDG 3), gender equality (SDG 5), and decent work (SDG 8).

## Methods and materials

### Study design

An exploratory-descriptive design was conducted from April–June 2023 to explore the lived experiences and perceptions of nurses who were mothers on exclusive breastfeeding. This design is appropriate when little is known about a phenomenon in a particular context, allows for the generation of richly detailed, context-sensitive findings, and investigates and portrays the real-life experiences of people, in contrast to depending on only perceived explanations of a specific event.

### Study setting

Savelugu Municipality in the Northern Region of Ghana, a predominantly agrarian Municipality with one main government hospital, several health centers, and Community-based Health Planning and Services (CHPS) compounds, was selected for its rural, significant health disparity, and distinct cultural and service contexts (dominant Muslim population, strong traditional practices) that may shape breastfeeding practices. In all, there are seventeen (17) Public Health Facilities in the Municipality. We purposely selected twelve out of the Seventeen Public Health Facilities for inclusion in the study because they were largely patronized.

### Sampling, recruitment, and saturation

#### Facility selection

Out of the 17 public health facilities in Savelugu Municipality, twelve were purposively selected for inclusion because they were the most patronized by mothers and provided a diverse representation of the municipality's healthcare services. Facilities not included were smaller, less frequented, or primarily offered limited maternal and child health services. This approach ensured that participants were recruited from settings where exposure to infant care and breastfeeding practices was most relevant.

#### Participant recruitment

Eligible participants were adult registered nurses (≥25 years) with at least three years of work experience, who had delivered a live infant within the previous two years, and had resided in the municipality for at least five years. Nurses meeting these criteria were identified in collaboration with facility in-charges. A total of 18 nurses were initially approached across the selected facilities: 15 agreed to participate, resulting in a response rate of 83%. Reasons for non-participation included scheduling conflicts and personal time constraints. Participants provided informed written consent before interviews commenced.

#### Operationalization of saturation

Data collection proceeded iteratively, with transcripts analyzed concurrently with ongoing interviews. Thematic/data saturation was considered achieved when no new codes, categories, or themes emerged from successive interviews. In this study, saturation was observed after 15 interviews, as the last three interviews yielded no additional themes beyond those already identified. This iterative approach, guided by Braun and Clarke's thematic analysis framework (25), ensured that the sample was sufficient to capture the breadth and depth of nurse-mothers’ experiences while minimizing redundancy.

Purposive selection of facilities and participants, coupled with iterative analysis, ensured that the study captured rich, contextually relevant insights. The transparent description of recruitment and saturation enhances the credibility, dependability, and transferability of the findings.

### Instrumentation and data collection

A semi-structured in-depth interview guide was used to explore nurse-mothers’ perceptions of exclusive breastfeeding (EBF), personal breastfeeding experiences, workplace supports and constraints, family dynamics, coping strategies, and recommendations to improve EBF practice. The guide was informed by prior literature on breastfeeding knowledge, practices, and support systems (26–29) to ensure coverage of key domains while allowing participants to freely express their experiences. The guide was piloted two months prior to data collection to refine questions for clarity and relevance.

#### Interviewers

All interviews were conducted in person by four trained female data collectors with master's degrees in public health and/or allied health research. All the data collectors had prior experience in qualitative research and in-depth interviewing with healthcare professionals. The interviewers were not employed in the facilities where participants worked, which minimized potential bias arising from pre-existing professional relationships. The first author supervised all data collection activities.

#### Positionality and power dynamics

The research team reflected on potential power imbalances between interviewers and participants, given differences in professional status and experience. Participants were reminded that participation was voluntary, that there were no right or wrong answers, and that responses would remain confidential. Open-ended questioning, active listening, and neutral prompts were used to reduce the influence of interviewer bias. Reflexive memos were maintained to document field observations, interactions, and possible biases, and these were discussed among team members during regular debriefing sessions.

#### Language considerations

Although interviews were conducted in English, the interviewers ensured that participants were fully comfortable expressing themselves in the language. Clarifications were provided in plain language as needed, and participants were encouraged to elaborate in their own words. All participants were able to communicate comfortably in English, and no translation into local languages was required.

#### Data collection procedures

Interviews lasted 45–60 min, were audio-recorded with participant consent, and transcribed verbatim. Interviews were conducted in private spaces convenient and comfortable for participants. Member checking was performed by replaying recordings and reviewing transcripts with participants to ensure accuracy. Additionally, the second and third authors independently validated transcripts and audio recordings, then reached consensus on emerging codes and themes. A third-party consultant further reviewed transcripts, coding, and themes to enhance trustworthiness.

#### Reflexivity

The research team engaged in ongoing reflexivity to examine how their professional backgrounds, assumptions, and prior experiences could shape data collection and interpretation. Reflexive discussions, peer debriefing, and audit trails were used to ensure transparency and minimize bias, with iterative review of transcripts to distinguish participants’ perspectives from researchers’ preconceptions. The main domains of the interview guide included:

Knowledge and perceptions of exclusive breastfeeding
Personal breastfeeding experiences and challenges
Workplace supports and constraints
Family and community support dynamics
Coping strategies and practical solutions
Recommendations to improve EBF practice

### Data analysis

All transcripts were analysed using thematic analysis following Braun and Clarke's (25) six-step framework. The analytic process was conducted systematically to enhance rigor, transparency, and confirmability.

#### Step 1: familiarization with data

The second and third authors independently read all transcripts multiple times to immerse themselves in the data and develop a holistic understanding of participants’ narratives. Field notes and reflexive memos were reviewed alongside transcripts to contextualize the responses.

#### Step 2: initial coding

Open coding was performed manually by the second and third authors. Codes were generated both inductively (emerging directly from participants’ words) and deductively (based on key domains from the interview guide: knowledge, experiences, workplace/family support, coping strategies, and recommendations). Each transcript was coded line-by-line, capturing both explicit statements and implicit meanings.

#### Step 3: code comparison and consensus

After initial coding, the two coders met to compare codes and discuss discrepancies. Differences in interpretation were resolved through discussion until consensus was reached. The first author acted as an arbiter in cases where agreement was not immediately possible.

#### Step 4: theme development

Codes were clustered into sub-themes and overarching themes through iterative review and discussion. Attention was paid to deviant or negative cases, i.e., participants whose experiences diverged from common patterns, to ensure that the analysis captured the full range of perspectives. Sub-themes were refined to ensure they were distinct, coherent, and representative of the data.

#### Step 5: third-party validation

A third-party qualitative research consultant independently reviewed transcripts, codes, and proposed themes. The consultant verified coding consistency, checked that sub-themes accurately reflected the raw data, and confirmed that deviant cases were adequately represented. This step enhanced the credibility and confirmability of the analysis.

#### Step 6: theme finalization and synthesis

Following validation, themes and sub-themes were finalized. Representative verbatim quotes were linked to each sub-theme to preserve the participants’ voices and demonstrate analytic transparency. Participant identifiers were replaced with pseudonyms (e.g., Participant 1) to maintain confidentiality.

### Demonstrating the analytic chain

An example of the coding and thematic process is provided in Table 1 below to illustrate the analytic chain from raw data to theme:

**Table 1:** Demonstration of analytic chain.

Data extract (verbatim)	Initial code	Sub-theme	Theme
“For me, I had a problem with the time at my work, it was difficult for me to do proper breastfeeding, so I stopped at four months afterwards…” (Participant 10)	Work time constraint	Time/workplace constraints	Perceptions of EBF
“I had limited support of my family members supporting me to do it…” (Participant 5)	Family support limited	Family support dynamics	Experiences of practicing EBF
“Maternity leave should be increased to 6/12 months post-delivery to make exclusive breastfeeding practical…” (Participant collective)	Longer maternity leave needed	Institutional recommendations	Coping strategies & recommendations

This step-by-step approach, combined with iterative discussion, reflexivity, and third-party validation, ensured that the analysis was systematic, credible, and transparent while reflecting the full range of participant experiences.

### Rigor and trustworthiness

The trustworthiness of the study was guided by Guba and Lincoln's (30) criteria, emphasizing credibility, transferability, dependability, and confirmability.

Credibility was enhanced through multiple strategies. Participants were invited to review interview summaries (member checking) to confirm that their perspectives were accurately captured. The research team engaged in prolonged engagement with transcripts and field notes to ensure a deep understanding of participants’ narratives. Although interviews were the primary data source, field notes from observations during interviews were used to contextualize participants’ verbal responses, providing additional insight into non-verbal cues and the interview environment.

Transferability was addressed by providing rich descriptions of the study setting, participant characteristics, and context, enabling readers to assess the applicability of findings to other rural or similar healthcare settings.

Dependability was maintained through a detailed audit trail documenting all coding decisions, theme development, and analytical discussions. Peer debriefing was conducted among the research team to reflect on interpretations and ensure analytical consistency.

Confirmability was strengthened through reflexive memoing, where researchers documented their assumptions, positionality, and reflections throughout data collection and analysis. To minimize single-observer bias and enhance the credibility of the analysis, we employed analyst triangulation. This involved a third-party qualitative consultant independently reviewing transcripts, codes, and themes to verify that the findings were grounded in the data. Additionally, methodological triangulation was pursued through the use of multiple validation strategies, including member checking, peer debriefing, and maintaining an audit trail, to cross-verify interpretations from the interview data.

### Ethical considerations

Ethical approval was obtained from the University for Development Studies Institutional Review Board (UDS/RB/260/2023). Participants gave informed written consent and were assured of confidentiality and voluntary participation. All methods were implemented in line with the Principles of the Declaration of Helsinki on ethical principles for medical research involving human subjects.

## Results

### Participant characteristics

A total of fifteen nurse-mothers participated in the study. Participants were aged between 25 and 38 years, with the majority (67%) aged 31–38 years. Most participants were married (67%) and had parity ranging from 0 to 4 children (87%). In terms of professional background, over half were Registered General Nurses (53%), with others comprising enrolled nurses, community health nurses, nurse practitioners, and specialist cadres. Participants’ years of professional experience ranged from 3 to over 10 years (Table 2).

**Table 2:** Participant characteristics.

Variable	Category	Frequency (*n*)	Percent (%)
Age	25–30 years	5	33
	31–38 years	10	67
Position	Nurse-In-Charge	1	7
	Staff/Senior Staff Nurse	2	13
	Junior/Senior/Principal Enrolled Nurse	5	33
	Specialist/Consultant Nurse	2	13
	Prescription Nurse	1	7
	Public Health Nurse (Community)	4	27
Cadre	Registered General Nurse	8	53
	Nurse Practitioner	1	7
	Nurse Specialist/Consultant	1	7
	Community Health Nurse	2	13
	Clinical Nurse Assistant	3	20
Qualifications	Bachelor's Degree	4	27
	Master's Degree	2	13
	Diploma	8	53
	Certificate	1	7
	PhD	0	0
	Membership/Fellowship	0	0
Marital Status	Single	5	33
	Married	10	67
Parity	0–4	13	87
	4+	2	13
Years of Experience	3–5 years	7	47
	5–10 years	5	33
	10+ years	3	20

### Thematic analysis overview

Three interrelated themes emerged from the analysis: (1) perceptions of exclusive breastfeeding (EBF), (2) experiences of practising EBF, and (3) coping strategies and recommendations.

Perceptions of EBF comprised five sub-themes: *knowledge of benefits; perceived impracticality and social restriction (“being socially tied down”); time and workplace constraints; privacy and workplace infrastructure; and information access and sharing*.

Experiences of practising EBF included four sub-themes: *EBF during maternity leave; return-to-work disruption and mixed feeding; breast milk expression challenges; and family support dynamics*.

Coping strategies and recommendations encompassed three sub-themes: *practical coping strategies; institutional recommendations; and community and family education*.

Table 3 presents a structured overview of themes, sub-themes, illustrative quotations, and interpretive meanings.

**Table 3:** Themes, sub-themes, illustrative quotes, and interpretation.

Main theme	Sub-theme	Illustrative quote (verbatim)	Interpretation
Perceptions of EBF	Knowledge of benefits	“At the hospital, pregnant women have access to breastfeeding information on how to do attachment and properly breastfeed … and this has helped me well in terms of breastfeeding”. (P8)	Participants demonstrate strong biomedical and practical knowledge of EBF, reflecting professional training and ANC exposure.
	Perceived social immobility and opportunity costs	“Breastfeeding is difficult to do because it socially ties you down… it affected me a lot”. (P6)	EBF is framed as restricting mobility, productivity, and social participation, revealing perceived opportunity costs beyond health knowledge.
	Time and workplace constraints	“I had a problem with the time at my work… so I stopped at four months”. (P10)	Shift work, workload, and limited maternity leave undermine sustained EBF.
	Privacy and workplace culture	“There is no private space, and sometimes you feel judged”. (P3)	Lack of infrastructure and unsupportive workplace norms discourage on-site breastfeeding or expression.
Experiences of practising EBF	EBF during maternity leave	“With my second child, I practised exclusive breastfeeding and it was good”. (P6)	Maternity leave provides a short window where EBF is feasible and positively experienced.
	Return-to-work disruption and mixed feeding	“I stopped exclusive breastfeeding at four months because I had to resume work”. (P9)	Returning to work marks a critical rupture point leading to early EBF cessation.
	Breast milk expression challenges	“Expressing the breast milk is very painful and sometimes it doesn't come”. (P4)	Physical discomfort, time pressure, and equipment access limit effective milk expression.
	Family support dynamics	“I had limited support of my family members supporting me to do it”. (P5)	Household norms and advice from relatives can undermine continued EBF.
Coping strategies and recommendations	Individual coping strategies	“You have to learn how to express the breast milk, if not it becomes very difficult”. (P10)	Nurses rely on personal coping strategies that partially compensate for structural gaps.
	Institutional recommendations	“Maternity leave should be increased to six months to make exclusive breastfeeding practical”. (Collective view)	Participants emphasise systemic policy reforms over individual responsibility.
	Family and community education	“When families understand the importance, it becomes easier for the mother”. (P8)	Extending education beyond mothers to families is seen as essential for sustained EBF.

### Narrative of themes and sub-themes

The themes presented below were generated through an iterative process of analytic synthesis. Initial open codes derived from participants’ narratives were progressively clustered into related categories and refined into sub-themes and overarching themes. While Table 3 summarises this process using illustrative quotations, the narrative that follows integrates and interprets these codes, situating participants’ experiences within their occupational, social, and institutional contexts.

#### Theme 1: perceptions of exclusive breastfeeding

##### Knowledge of benefits and professional awareness

Participants consistently demonstrated strong knowledge of EBF and articulated positive attitudes toward the practice. Many described EBF as “the baby's first medicine” and emphasised its role in promoting infant health and immunity. This knowledge was largely attributed to professional training and exposure through antenatal care (ANC) services and routine clinical practice. As one participant noted:

“At the hospital, pregnant women have access to breastfeeding information on how to do attachment and properly breastfeed … and this has helped me well in terms of breastfeeding”. (Participant 8)

These accounts suggest that formal information systems and professional education around EBF were functioning effectively and that nurse-mothers possessed the technical and biomedical understanding required to practise and promote EBF.

##### Perceived impracticality and social restriction

Despite recognising the benefits of EBF, many participants perceived it as socially and practically restrictive. Several nurses described EBF as “socially tying you down,” limiting their mobility, flexibility, and ability to meet work and family obligations. One participant explained:

“Breastfeeding is difficult to do because it socially ties you down. I had a lot of challenges with breastfeeding, so I didn’t express much breastmilk, and this affected me a lot”. (Participant 6)

This sub-theme captures a perceived tension between the ideals of EBF and the lived realities of working motherhood. Nurses framed EBF not as undesirable, but as difficult to sustain within the demands of paid work, household responsibilities, and social expectations.

##### Time and workplace constraints

Time scarcity and workplace demands were repeatedly cited as major barriers to sustained EBF. Participants described long shifts, rigid schedules, staff shortages, and high patient loads as incompatible with regular breastfeeding or milk expression. One nurse summarised this challenge as follows:

“For me, I had a problem with the time at my work; it was difficult for me to do proper breastfeeding, so I stopped at four months afterwards”. (Participant 10)

These accounts highlight how occupational structures, particularly shift work and limited control over time, constrained nurses’ ability to align EBF recommendations with their professional responsibilities.

##### Privacy and workplace infrastructure

Concerns about privacy and workplace culture further shaped nurses’ perceptions of EBF. Many participants reported discomfort breastfeeding or expressing milk at work due to the absence of private spaces and fear of judgement from colleagues or patients. As one participant stated:

“Breastfeeding at work feels uncomfortable; there is no private space, and sometimes you feel judged”. (Participant 3)

The lack of dedicated lactation rooms or supportive infrastructure contributed to feelings of embarrassment and reluctance to breastfeed at work, reinforcing early discontinuation of EBF even among motivated mothers.

##### Information access and sharing

Participants consistently reported adequate access to EBF information through ANC services and health facility-based education. However, their narratives underscored a clear distinction between being informed and being able to practise EBF. Although information flow was described as robust, knowledge alone was insufficient to overcome structural and social barriers. This gap highlights the limits of education-focused interventions in the absence of enabling workplace and family environments.

#### Theme 2: experiences of practising exclusive breastfeeding

##### EBF during maternity leave

Most participants reported initiating and practising EBF during maternity leave and described this period positively. For many, maternity leave represented the only phase in which EBF was practically feasible. One participant reflected:

“I personally experienced breastfeeding with my second child, and I think it is good”. (Participant 6)

These accounts suggest that intention and adherence to EBF were highest when work-related constraints were temporarily removed.

##### Return-to-work disruption and mixed feeding

A dominant pattern across narratives was the disruption of EBF upon returning to work. Participants commonly reported introducing mixed feeding or discontinuing EBF entirely once maternity leave ended. One nurse explained:

“I stopped exclusive breastfeeding at four months because I had to resume work and there was no one to bring the baby to me”. (Participant 9)

The short statutory maternity leaves in Ghana (approximately 12 weeks), combined with shift work and limited childcare options, emerged as a critical rupture point in EBF trajectories.

##### Breast milk expression challenges

Breast milk expression was frequently described as painful, time-consuming, and unpredictable. Some participants struggled with low milk output, while others relied on mechanical pumps where available. As one nurse noted:

“The experience I had expressing the breast milk … is that it is very painful and sometimes it does not even come regularly”. (Participant 4)

These challenges reduced the feasibility of sustaining EBF after returning to work, particularly in settings without adequate time allowances, equipment, or technical support.

##### Family support dynamics

Family support played a significant but variable role in shaping EBF experiences. Some participants reported limited assistance from partners or extended family members, while others described pressure from relatives to introduce complementary foods early. As one participant stated:

“I think my experience with exclusive breastfeeding is that I had limited support of my family members supporting me to do it”. (Participant 5)

These dynamics illustrate how household norms and intergenerational expectations intersected with workplace constraints to influence feeding decisions.

#### Theme 3: coping strategies and recommendations

##### Practical coping strategies

In response to these challenges, participants adopted a range of coping strategies, including manual or mechanical expression, night-time feeding, and relying on informal support from colleagues. While these strategies enabled temporary continuation of breastfeeding, participants framed them as exhausting and unsustainable rather than long-term solutions.

##### Institutional recommendations

Participants strongly emphasised the need for organisational and policy-level changes. Key recommendations included extending maternity leave to six months, providing lactation rooms and protected expression breaks, introducing workplace childcare or crèches, and fostering supportive workplace cultures. These recommendations reflect nurses’ recognition that individual effort alone cannot overcome structural barriers.

##### Community and family education

Finally, participants highlighted the importance of involving partners, family members, and community leaders in breastfeeding education. They argued that strengthening household-level support could reduce social pressure on nurse-mothers and align family expectations with EBF recommendations.

##### Key analytic insights

A key cross-cutting insight from the analysis is the tension inherent in nurses’ dual role as both health educators promoting EBF and mothers attempting to practice it. While participants possessed professional authority and routinely counselled mothers on exclusive breastfeeding, their own experiences were marked by compromise, adaptation, and early discontinuation. This disjuncture did not undermine their belief in EBF, but rather heightened awareness of the gap between ideal recommendations and structural realities. Nurses lived experiences thus illuminate how professional knowledge coexists with constrained practice, especially within resource-limited and gendered work environments.

The findings suggest three analytically relevant propositions that may inform future hypothesis-driven research:

(1). High professional knowledge of EBF does not translate into sustained practice in the absence of organizational and policy support.
(2). Return to work functions as a critical structural point in EBF trajectories among nurse-mothers.
(3). Nurses’ personal breastfeeding experiences shape how they interpret, contextualize, and emotionally engage with EBF counselling, even when formal guidelines remain unchanged.

This dual-role tension highlights the limits of education-based interventions in the absence of supportive work and family environments, particularly in low-resource and rural health system contexts.

## Discussion

This study explored nurse-mothers’ perceptions and lived experiences of exclusive breastfeeding in Savelugu Municipality, revealing a complex but instructive picture. Nurses demonstrated strong knowledge and positive attitudes toward EBF, often drawing from their personal experiences. However, their ability to sustain EBF for the recommended six months was significantly undermined by structural, social, and occupational barriers. These findings provide important insight into how knowledge does not always translate into sustained practice, a gap with implications for public health policy, workplace support systems, and clinical practice. Beyond individual-level challenges, participants’ accounts reveal how occupational structures shape breastfeeding outcomes. Shift-based work, unpredictable schedules, staff shortages, and high patient load limited nurses’ autonomy over time and space, making EBF difficult to sustain even when motivation was high. These constraints were particularly salient in rural facilities where staffing gaps intensified workloads. The findings suggest that early cessation of EBF among nurse-mothers is less a matter of individual choice and more a consequence of institutional work organization and health system constraints. From a conceptual standpoint, these experiences can be interpreted through the lens of work–family conflict theory, highlighting the tension between professional responsibilities and caregiving demands. They also underscore the importance of organisational support for breastfeeding and reflect gendered labour perspectives, where caregiving responsibilities disproportionately impact women even within professional roles.

Most participants demonstrated a clear understanding of the benefits of EBF, describing it as “very good” and “the baby's first medicine”. Many had practiced EBF at least partially, with one respondent stating, “I had practiced exclusive breastfeeding with my first baby”. This finding aligns with prior studies reporting that nurses are often active practitioners of EBF, serving as positive role models for mothers in their care (31). However, our findings contrast with Ansu-Mensah et al. (32), who reported low EBF practice rates among nurses in southern Ghana. The difference may be attributable to contextual variations, for example, nurses in rural northern Ghana may face fewer competing private-sector job pressures but more sociocultural influences, which could shape their motivation and attitudes. This study extends existing research by theorizing nurses’ breastfeeding experiences not merely as individual challenges, but as manifestations of a broader dual-role paradox in which professional knowledge coexists with structurally constrained maternal practice. Also, these findings highlight the potential for leveraging nurse-mothers as champions of EBF through structured peer-support programs. At the facility level, out findings suggest that their involvement could be formalized into workplace health promotion initiatives. Clinically, our findings suggest that nurses who practice EBF could be better positioned to deliver empathetic, practical counselling to new mothers.

Participants reported that information flow on EBF was robust during antenatal care (ANC) visits and within health facilities: “There is always information flow when I go to ANC”. This indicates that knowledge dissemination systems are functional. However, despite this high level of awareness, EBF discontinuation before six months was common. This finding contrasts with studies that reported limited breastfeeding information among nurses in other settings, suggesting that knowledge alone is not sufficient to guarantee adherence (26, 33). The gap highlights the need for structural enablers like maternity leave, workplace support, and family engagement to complement education. Our findings suggest that EBF education programs could integrate family and community stakeholders, especially influential relatives such as grandmothers and partners, to create a shared understanding of EBF benefits. At the public health practice level, our findings suggest that integrating male-partner and family education sessions into ANC/PNC could strengthen social support. Clinically, our findings suggest that providers could adopt a family-centred counselling approach to address potential resistance to EBF from household members.

A major barrier to sustained EBF was the short statutory maternity leave (typically 12 weeks) and inflexible work schedules, which forced many nurses to introduce mixed feeding before six months. As one participant noted, “I stopped exclusive breastfeeding at four months because I had to resume work and there was no one to bring the baby to me”. This aligns with Emagneneh et al. (34), who identified workplace constraints as a key predictor of early cessation of breastfeeding. Participants also expressed dissatisfaction with the lack of private spaces for expressing or feeding at work: “Breastfeeding at work feels uncomfortable; there is no private space”. This differs from Fatima et al. (35), who reported that nurses in their study settings had adequate privacy provisions, underscoring variability across facilities. Our findings suggest the need to extend paid maternity leave to six months in line with WHO recommendations. Our findings suggest that facility-level interventions such as workplace lactation rooms, designated breaks for expression, and on-site crèche services could create an enabling environment. Clinically, our results suggest that occupational health units could incorporate breastfeeding support into staff welfare programs to mitigate early discontinuation.

Despite awareness and willingness, some participants reported difficulty with expressing milk, describing it as painful and time-consuming: “The experience I had expressing the breast milk … is that it is very painful and sometimes does not even come regularly”. This finding echoes Shahrani et al. (36), who found that inadequate technical support for milk expression contributes to early cessation. Pain, low supply, and lack of equipment may reduce mothers’ ability to sustain EBF after returning to work. Our findings suggest support such as subsidized or employer-provided breast pumps and training on expression techniques. At the facility level, our findings highlight that lactation consultants or trained midwives could provide hands-on demonstrations and follow-up. Clinically, our results suggest that postpartum counselling could include troubleshooting for expression difficulties and early referral to lactation support when needed.

Moreover, several participants highlighted the influence of family members on infant feeding decisions. Some reported limited support, while others noted pressure from relatives to introduce complementary foods early. This finding aligns with Dukuzumuremyi et al. (37), who emphasized the central role of grandmothers and partners in shaping EBF outcomes. These social pressures may explain why, despite high knowledge levels, some nurses were unable to sustain EBF for the full six months. Hence, our findings suggest that public health programs could adopt a household-centred approach to breastfeeding promotion. Our findings also suggests that campaigns could target family decision-makers to address harmful norms. Clinically, our results highlight that nurses could use ANC classes to engage entire households and encourage shared responsibility for supporting breastfeeding mothers.

An important analytic insight emerging from this study concerns the nature and meaning of the coping strategies adopted by nurse-mothers. Participants described strategies such as expressing breast milk, night-time feeding, and reliance on informal family or collegial support as necessary responses to competing work and caregiving demands. Crucially, however, these strategies were not framed by participants as viable or empowering long-term solutions. Rather, they were consistently described as exhausting, stressful, and ultimately unsustainable. Analytically, these practices could therefore be understood not as evidence of individual resilience or successful adaptation, but as short-term, emergency responses to structural and institutional constraints, including inadequate maternity leave, inflexible work schedules, and the absence of workplace lactation infrastructure. Making this distinction is important to avoid an unintended individualization of responsibility, whereby the burden of sustaining exclusive breastfeeding is implicitly shifted onto mothers rather than addressed at the level of policy, organizational design, and social support systems.

A central analytic contribution of this study is the paradox between high professional knowledge of EBF and the structural, technical, and social barriers that limit its practice. While nurse-mothers demonstrated strong understanding of EBF benefits and often had positive personal experiences, many were unable to sustain exclusive breastfeeding for the recommended six months. Structural constraints, including short statutory maternity leave, inflexible work schedules, and lack of private spaces for expression or feeding, combined with technical difficulties and social pressures from family members, created significant barriers. Interpreted through work–family conflict theory, these findings reveal how occupational demands can compete with caregiving responsibilities. From an organisational support perspective, the results underscore the need for workplace infrastructure and policy interventions. Viewed through a gendered labour lens, the study highlights how women disproportionately bear the burden of balancing professional duties and reproductive responsibilities. This paradox highlights that knowledge alone is insufficient to ensure adherence, underscoring the need for interventions that address workplace infrastructure, family support, and practical challenges, in addition to continued education.

### Ethical considerations of choice, autonomy, and professional responsibility

While exclusive breastfeeding is strongly promoted in public health policy and clinical guidelines, breastfeeding decisions are shaped by structural, occupational, and personal constraints. An important ethical consideration emerging from this study concerns how nurse-mothers, as health educators, balance evidence-based promotion of EBF with respect for maternal autonomy and empathy for women facing genuine barriers. Participants’ experiences highlight the tension between professional expectations to promote EBF and lived realities that make sustained EBF difficult, even for health professionals themselves. This underscores the ethical imperative for breastfeeding counselling that is supportive rather than prescriptive, acknowledges contextual constraints, and respects informed choice. Recognising these ethical dimensions strengthens the interpretation of the findings and reinforces the need for counselling approaches and workplace policies that support, rather than moralise, breastfeeding decisions.

### Strengths and limitations

A major strength of this study is its focus on nurse-mothers as both healthcare providers and primary caregivers; a group whose dual role uniquely positions them as role models and promoters of exclusive breastfeeding. The study's exploratory-descriptive design allowed for a nuanced understanding of perceptions and lived experiences, capturing the complexities of balancing professional duties and breastfeeding demands. The inclusion of verbatim quotes also amplified participants’ voices, grounding the analysis in lived realities and allowing for direct policy and practice implications to be drawn.

However, the study has some limitations that should be acknowledged. First, the use of a single-site design in Savelugu Municipality may limit the transferability of findings to other regions in Ghana or beyond, where workplace policies, cultural norms, and health system capacities may differ. Second, because the study relied on self-reported experiences, there is the possibility of recall bias or social desirability bias, especially given that nurses are trained to promote EBF and might overstate their adherence to recommended practices.

Third, this study relied on retrospective accounts of exclusive breastfeeding experiences, with some participants reflecting on events that occurred up to two years prior to the interviews. As with all retrospective qualitative research, participants’ narratives may have been shaped by recall bias, *post-hoc* rationalization, or professional norms acquired through continued clinical practice. Nurses’ evolving professional identities and accumulated counselling experience may have influenced how past breastfeeding decisions were interpreted and narrated. To mitigate these effects, interviews encouraged concrete descriptions of events, timelines, and decision points rather than general evaluations, and probes were used to elicit specific examples. During analysis, attention was paid to internal consistency across narratives and to identifying shared patterns rather than treating individual accounts as objective reconstructions of past behavior. Nonetheless, findings should be interpreted as reflective accounts rather than real-time observations of practice.

Fourth, while the qualitative approach provides depth, it does not allow for quantitative measurement of the prevalence of EBF adherence among nurses, which could be addressed in future mixed-methods research. Finally, the perspectives of other key stakeholders, such as partners, grandmothers, and facility administrators, were not included, yet these groups strongly influence EBF decisions and could have provided a more holistic view of systemic barriers and facilitators.

Despite these limitations, this study uniquely highlights the paradox between strong knowledge of exclusive breastfeeding and the structural and social barriers that limit sustained practice. The study provides context-specific insights from a rural, low-resource setting in northern Ghana, where data on working mothers’ breastfeeding experiences are limited.

### Implications for Policy, Practice, and Research

The evidence highlights the need to strengthen both national and institutional maternity protection frameworks. Short maternity leave and heavy workloads continue to undermine EBF, suggesting that government agencies such as the Ministry of Health (MoH), Ministry of Employment and Labour Relations, and Parliament could consider progressively extending maternity leave toward the WHO-recommended six months. In the shorter term, health institutions and employers can implement more immediately feasible measures, such as protected breastfeeding or milk expression breaks, flexible scheduling, safe lactation spaces, and on-site childcare (crèches). Labour unions and professional nursing associations can advocate for these changes and monitor compliance, while international partners (WHO, UNICEF, ILO) could offer technical guidance to align policies with global standards.

At the institutional level, sustaining EBF requires coordinated efforts within hospitals and families. Nurses and midwives, despite their knowledge and positive attitudes, often face structural barriers that limit EBF practice. Hospital management and nursing directors can adopt policies that normalize breastfeeding breaks, provide stigma-free spaces for expression, and offer flexible work arrangements to accommodate new mothers. Health educators and midwives could include partners and family members in antenatal and postnatal counselling, fostering supportive home environments. Community health volunteers and local leaders can reinforce household and societal support through education campaigns on shared responsibility for breastfeeding.

Future research could engage diverse stakeholders to identify feasible, context-specific interventions. Universities and nursing research institutions can lead longitudinal and intervention studies evaluating the impact of extended maternity leave, flexible work schedules, and workplace infrastructure on EBF outcomes. Policy think tanks and public health research centres can assess the cost-effectiveness of interventions such as crèches, lactation rooms, and workplace expression support. Professional bodies, including the Ghana Registered Nurses and Midwives Association (GRNMA), can collaborate with researchers to generate evidence on healthcare workers’ experiences balancing professional duties and breastfeeding. Partnerships with development partners (UNICEF, WHO, NGOs) could also support operational research on affordable, user-friendly breastmilk expression technologies suitable for low-resource settings.

The findings also point to coping as a potentially fruitful focus for future research. Rather than treating coping strategies as neutral or adaptive behaviors, future qualitative and mixed-methods studies could place coping at the center of analysis to distinguish between different forms of coping (e.g., compensatory, sacrificial, collective) and to assess their physical, emotional, and professional costs over time. Such work could help clarify which strategies merely mask systemic shortcomings and which, if any, might be supported or scaled up without reinforcing unsustainable expectations placed on working mothers. This line of inquiry would further illuminate how health systems implicitly rely on women's unpaid labor and endurance to compensate for institutional gaps.

## Conclusions

This study highlights the paradox of high EBF knowledge but low adherence beyond three to four months among nurse-mothers in rural northern Ghana. Structural barriers, including short maternity leave, lack of workplace lactation infrastructure, and inadequate privacy, coupled with technical challenges and social pressures, undermine sustained EBF practice. Our findings suggest that strengthening workplace and family support systems could be critical to bridging this knowledge-practice gap.

## Data Availability

The raw data supporting the conclusions of this article will be made available by the authors, without undue reservation.
